# Effect of Three Different Grip Angles on Physiological Parameters During Laboratory Handcycling Test in Able-Bodied Participants

**DOI:** 10.3389/fphys.2015.00331

**Published:** 2015-11-23

**Authors:** Thomas Abel, Brendan Burkett, Barbara Thees, Stefan Schneider, Christopher D. Askew, Heiko K. Strüder

**Affiliations:** ^1^Institute of Movement and Neurosciences, German Sport University CologneCologne, Germany; ^2^Faculty of Science, Health, Education and Engineering, School of Health and Sport Sciences, University of the Sunshine CoastMaroochydore, QLD, Australia

**Keywords:** adapted physical activity, biomechanics, spinal cord injury

## Abstract

**Introduction:** Handcycling is a relatively new wheelchair sport that has gained increased popularity for people with lower limb disabilities. The aim of this study was to examine the effect of three different grip positions on physical parameters during handcycling in a laboratory setting.

**Methods:** Twenty one able-bodied participants performed three maximum incremental handcycling tests until exhaustion, each with a different grip angle. The angle between the grip and the crank was randomly set at 90° (horizontal), 0° (vertical), or 10° (diagonal). The initial load was 20 W and increased by 20 W each 5 min. In addition, participants performed a 20 s maximum effort.

**Results:** The relative peak functional performance (W/kg), peak heart rate (bpm), associated lactate concentrations (mmol/l) and peak oxygen uptake per kilogram body weight (ml.min^−1^.kg^−1^) for the different grip positions during the stage test were: (a) Horizontal: 1.43 ± 0.21 W/kg, 170.14 ± 12.81 bpm, 9.54 ± 1.93 mmol/l, 30.86 ± 4.57 ml/kg; (b) Vertical: 1.38 ± 0.20 W/kg, 171.81 ± 13.87 bpm, 9.91 ± 2.29 mmol/l, 29.75 ± 5.13 ml/kg; (c) Diagonal: 1.40 ± 0.22 W/kg, 169.19 ± 13.31 bpm, 9.34 ± 2.36 mmol/l, 29.39 ± 4.70 ml/kg. Statistically significant (*p* < 0.05) differences could only be found for lactate concentration between the vertical grip position and the other grips during submaximal handcycling.

**Conclusion:** The orientation of three different grip angles made no difference to the peak load achieved during an incremental handcycling test and a 20 s maximum effort. At submaximal load, higher lactate concentrations were found when the vertical grip position was used, suggesting that this position may be less efficient than the alternative diagonal or horizontal grip positions.

## Introduction

Handcycling has opened a new world of mobilization for people who are restricted to a wheelchair, from both a health perspective (Abel et al., 2003a; Arnet et al., 2016) and for sports performance (Abel et al., 2006; Goosey-Tolfrey et al., 2006; de Groot et al., 2014). During the last 5 years, race performance has increased significantly with the adoption of elite athlete training approaches and technical developments concerning the handcycle itself. In comparison to wheelchair propulsion, handcycling has a higher mechanical efficiency (Abel et al., 2003a; Dallmeijer et al., 2004; Simmelink et al., 2015; Arnet et al., 2016), which gives the person restricted to a wheelchair the benefit of increased mobility. It has been postulated that regular engagement with handcycling will likely lead to fewer painful and debilitating overuse injuries (van der Woude et al., 2006; Arnet et al., 2014). Energy expenditure in handcycling is sufficient to offer protection against the development of secondary conditions such as cardiovascular disease (Abel et al., 2003a; van der Woude et al., 2013). As a relatively new device there have been a range of areas investigated to improve handcycle performance, such as the influence of back rest position, gear ratios (Faupin et al., 2008; Arnet et al., 2014). Whilst the efficiency of the athlete and handcycle as a complete system has been assessed, the influence of some key components within this system have not yet been quantified, such as the type or orientation of the hand grip.

As a mechanical device, the transmission of force from the athlete to the cycle plays a major role in handcycling performance. To better understand this interface between the athlete and the cycle, the influence of crank length (Goosey-Tolfrey et al., 2008; Krämer et al., 2009) and crank patterns (Verellen et al., 2004, 2008) on the transmission of forces has been investigated. In fine tuning this connection further, the configuration of cranking, either synchronous or asynchronous, has also been investigated (Hopman et al., 1995; Mossberg et al., 1999; Abel et al., 2003b; Dallmeijer et al., 2004; Goosey-Tolfrey and Sindall, 2007; van der Woude et al., 2008). To date the research on crank configurations has failed to address the critical question of hand-crank grip position. From a purely anatomical perspective, the musculoskeletal structure of the human forearm is a significant determinant of the ergonomics of the wrist, with the maximum generation of force found when the wrist is orientated near maximum flexion (Morse et al., 2006; Khan et al., 2009). Due to their disability, the users of a handcycle often have some degree of movement limitation in their forearm, therefore the optimisation of grip position for these athletes is of great importance (Bressel et al., 2001). In practice, disabled athletes commonly self-experiment with different grip angels and different grip forms. To investigate the optimal grip-crank interface, the aim of this study was to examine the effect of three different grip angles on the physiological responses to incremental and maximal handcycling in a laboratory setting. The hypothesis herby is that altering the grip orientation, and therefore altering the muscle length and specific load applied to the forearm and upper muscles, will result in changes change in power generated as well as changes in physiological reactions at submaximal and maximal load.

## Methods

### Participants

Twenty-one participants (15 male and six female; age 27 ± 5 years, height 178.0 ± 11.9 cm, weight 74.7 ± 13.3 kg) performed three stage tests until exhaustion with different grip angles. The participants were able-bodied and with a good training status of the upper extremity (active athletes in swimming and triathlon).

This study was carried out in accordance with the recommendations of guidelines of the International Committee of Medical Journal Editors. All subjects gave written informed consent in accordance with the Declaration of Helsinki. All investigations were approved by the German Sports University ethical advisory committee.

### Experimental overview

**Figure 1:**
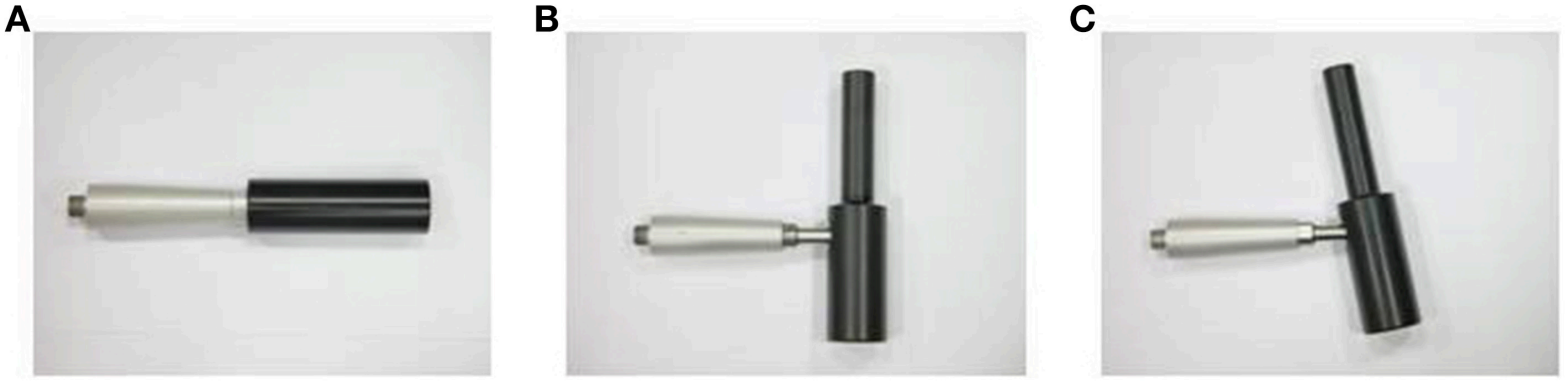
**Angle between the grip and the crank (A) 90° (horizontal) (B) 0° (vertical) (C) 10° (diagonal)**.

For all tests, participants sat in an arm-power race handcycle (Sopur Shark, Sunrisemedical Germany) connected to an ergometer (Cyclus II, Richter; Germany). The crank length was 175 mm, backrest angle approximately 45° adapted to the participants to avoid full elbow extension during crank revolutions. Crank housing position was set on a horizontal line to shoulder angle, crank configuration synchronous. The Cyclus II ergometer has been validated as an accurate measure of handcycling work load (Reiser et al., 2000). The angle between the grip and the crank was set in one of three configurations (see Figure 1), (a) 90° (horizontal = H), (b) 0° (vertical = V) and (c) 10°with respect to the vertical (i.e., diagonal, common way of cranking = D). Participants conducted an incremental test and a 20-s peak force test. The 20-s peak test was carried out after the incremental test und separated by 2 h. Tests were repeated three times using each of the grip configurations, in a random order, and each testing session was separated by a 3-day recovery period.

### Incremental test protocol

After a standardized warm up period, the participants commenced hand cycling using one of the defined grip positions. Cycling cadence was freely chosen above 50 rpm. The initial load during test was 20 W and increased by 20 W every 5 min until the load where the 50 rpm cadence was not able to be maintained.

Expired air was collected continuously (ZAN 600, ZAN, Germany) during exercise for the assessment of oxygen consumption. Immediately before every test session, gas analyzers were calibrated with known reference gas mixtures (room air and a standard certified commercial gas preparation). The expiratory airflow volume was calibrated using a 1.0-l syringe. Blood samples to determine lactate concentrations were taken from the earlobe during the last 30 s of each stage (Biosen C, Eppendorf, Germany). Heart rate was monitored continuously (Polar X-Trainer, Polar, Finland).

### Data analysis

The descriptive mean and standard deviations for each of the measures of work, heart rate, blood lactate, and oxygen consumption were calculated using STATISTICA for Windows Version 7.1 F (StatSoft Inc, Tulsa, USA). An analysis of variance with repeated measurements for submaximal and peak values was used to determine the presence of TIME and GRIP effects for heart rate, lactate and VO_2peak_. *Post-hoc* (least significant difference Test LSD) analysis was performed where there were significant main effects and interactions to determine the precise location of differences or changes. A *P*-value less than 0.05 was considered significant.

## Results

The peak functional performance (W/kg), peak heart rate (bpm), the associated lactate concentrations (mmol/l), and peak oxygen uptake per kilogram body weight (ml/kg) for the three different grip positions during handcyling are shown in Table 1.

**Table 1:** **Physiological values at peak load during the stage test**.

	**Horizontal**	**Vertical**	**Diagonal**
Relative work load (W/kg)	1.43 ± 0.21	1.38 ± 0.20	1.40 ± 0.22
Heart rate (bpm)	170.14 ± 12.81	171.81 ± 13.87	169.19 ± 13.31
Lactate (mmol/l)	9.54 ± 1.93	9.91 ± 2.29	9.34 ± 2.36
Relative oxygen uptake (ml/kg)	30.86 ± 4.57	29.75 ± 5.13	29.39 ± 4.70

**Figure 2:**
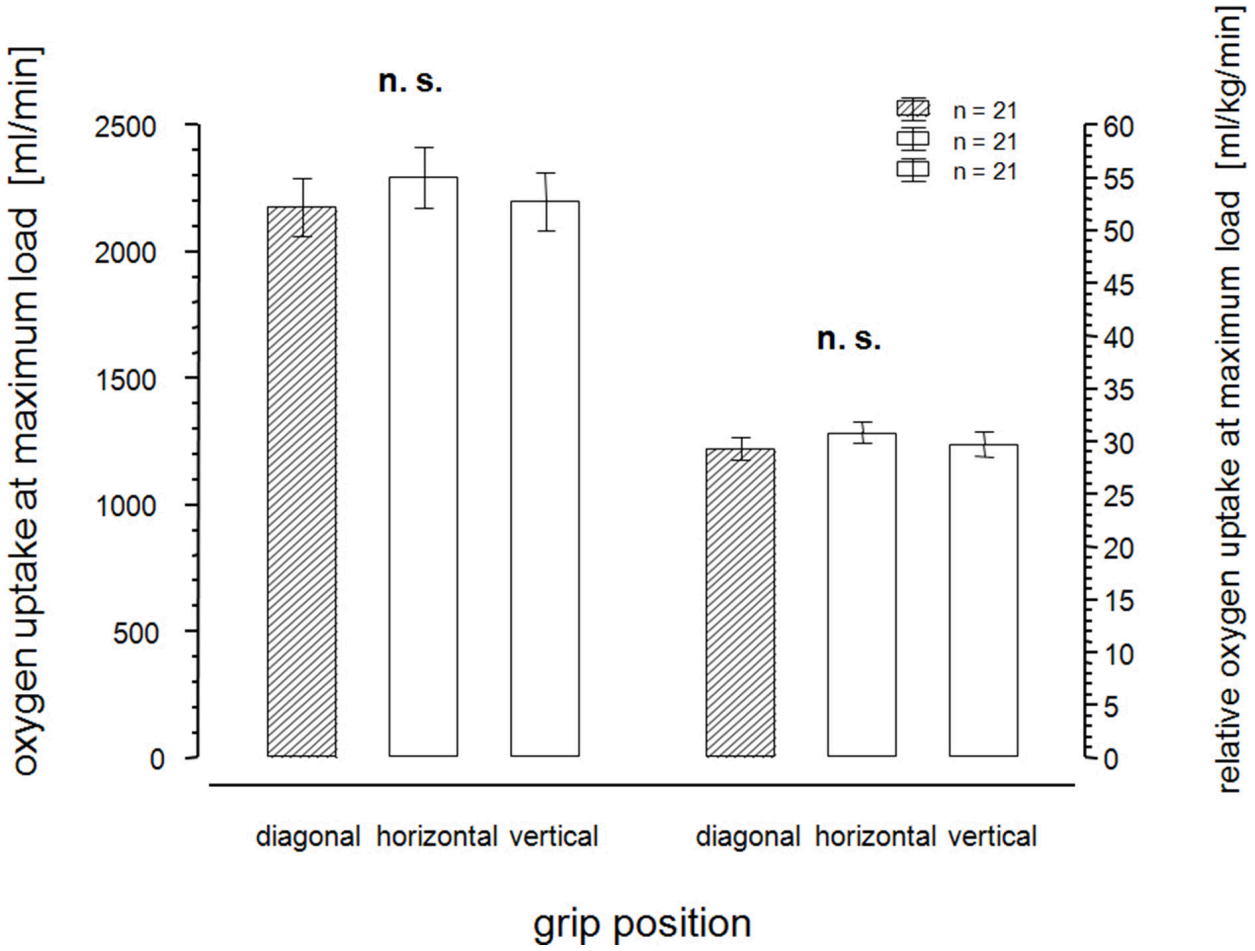
**Absolute and relative oxygen uptake at maximum load**.

**Figure 3:**
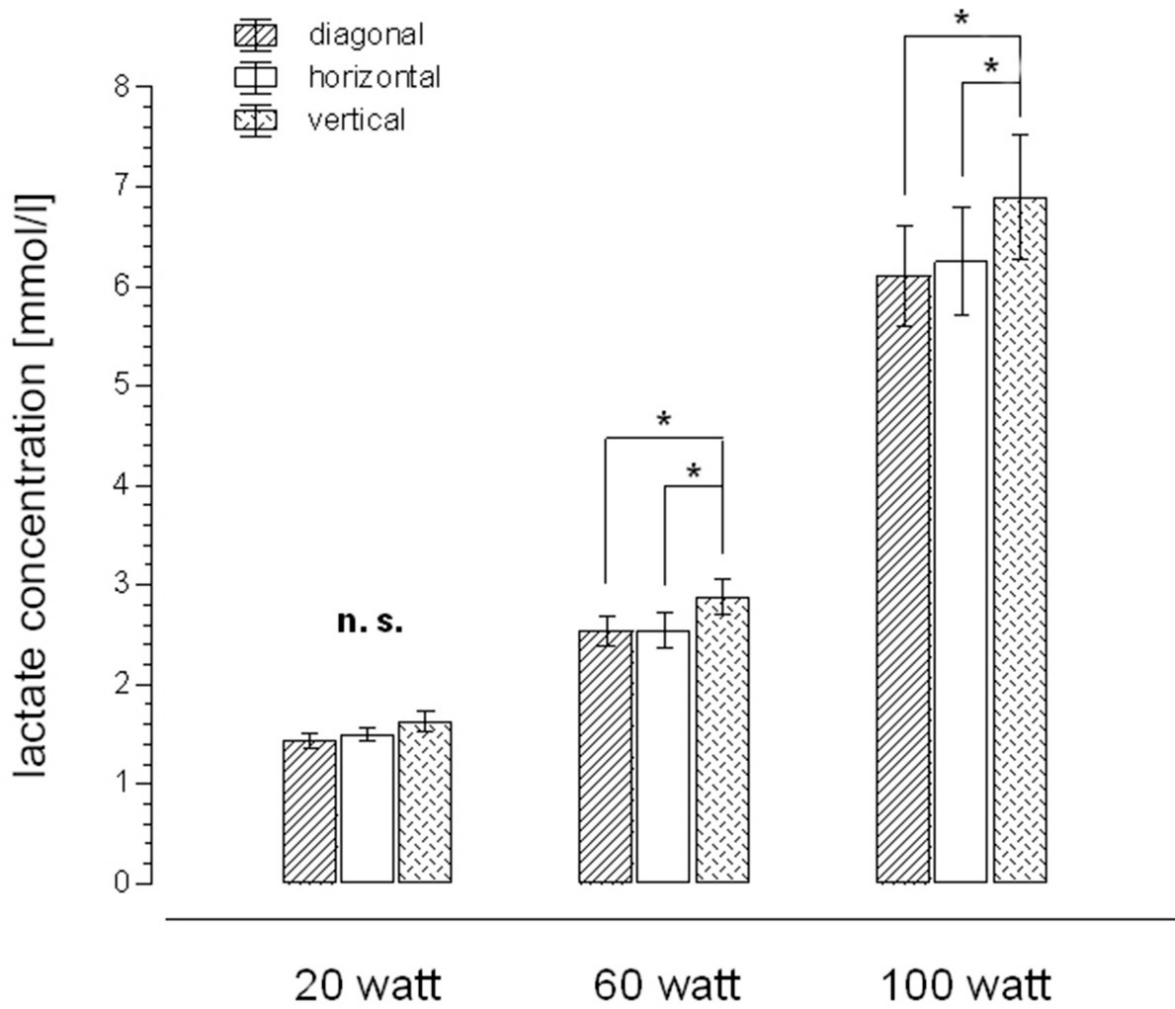
**Lactate concentrations at defined work loads of 20, 60, and 100 W for different grip angles**. ^*^Significant.

As shown in Figure 2, there were no significant differences for peak oxygen uptake between the three grip positions during the incremental test. There were also no differences between the three grip positions for the other peak variables during handcycling, including functional performance, heart rate and blood lactate. Figure 3 shows the lactate concentrations at defined submaximal work loads of 20, 60, and 100 W during the incremental test watts. There was a statistically significant difference between the vertical grip position and the other grips at 60 and 100 W.

Peak and average data for each of the variables during the 20 s all out test are shown in Table 2. No significant differences were found between the three gips positions.

**Table 2:** **Peak and mean work load during the 20 s test**.

	**Horizontal**	**Vertical**	**Diagonal**
Peak load (W)	589.73 ± 190.78	581.52 ± 188.24	583.67 ± 211.75
Relative peak load (W/kg)	7.74 ± 1.51	7.64 ± 1.59	7.59 ± 1.76
Mean load 20 s. (W)	350.17 ± 125.75	341.97 ± 116.18	344.02 ± 128.35
Mean relative load 20 s. (W/kg)	4.57 ± 1.08	4.48 ± 0.98	4.47 ± 1.10

## Discussion

The aim of this study was to examine the effect of three different grip angles on functional performance and associated physiological variables during handcycling. To the authors' knowledge, this aspect of handcycling has not been previously investigated.

While absolute an relative oxygen uptake tended to be lower at submaximal and peak workloads when the diagonal grip orientation was used, this was not statistically significant. Nonetheless, small reductions in oxygen uptake during laboratory tests may translate into important and significant improvements in economy during longer endurance activities such as a handcycling road race (Fischer et al., 2015). This time dependant relationship between work load and oxygen uptake has been identified in other studies (Verellen et al., 2004).

An unexpected finding was the higher blood lactate concentration during submaximal (60 and 100 W) handcycling when the vertical grip was used compared with both the diagonal and horizontal grips. As it is unlikely that lactate clearance would be different between the three test conditions (Heck et al., 1985; Mader, 2003), this elevation in lactate with the vertical grip indicates that there is likely to be a greater reliance on anaerobic metabolism by the working muscles. As these changes are unique to the vertical grip position, a plausible hypothesis could be that the vertical position requires increased static work, throughout the entire pedal stroke, to fix the hand at the handlebar. As sweat production, and the associated grip instability, increases with exercise intensity and time, this is likely to lead to further increases in static work and a greater reliance on local anaerobic metabolism. It is likely that this explains why many athletes avoid using the vertical grip in practice, and instead adopt a grip with some degree of horizontal orientation.

In the present study a full 90° grip range was explored to ascertain the most appropriate orientation of the hand-crank grip. This complete range of movement was considered necessary, as previous cycling studies on crank length for example only considered small increments of change (Martin and Spirduso, 2001). Despite this maximum change in the range of motion of the grip orientation, there were no significant differences in force between the vertical, diagonal, and horizontal grip positions. The hypothesis that altering the grip orientation, and therefore altering the muscle length and specific load applied to the forearm and upper muscles, would result in a change in power generated as well as in efficiency related values was not supported. Based on the similar oxygen uptake and heart rate measures during each of the tests, the economy of the three different grip orientations showed no difference.

Training with the optimal hand-crank orientation is essential for efficiency of movement, performance and the prevention of overuse risks (Webborn and Van de Vliet, 2012; Arnet et al., 2014). As the economy of movement when handcycling with the diagonal grip was only slightly, and non-significantly, higher than the other grip orientations for the able-bodied population, it would also seem important to consider comfort when setting up the handcycle, particularly for individuals with a loss of lower limb function. Depending on the unique and individual anatomy and movement restrictions of the athlete with a disability, the optimal handcycle setup and grip orientation may alter significantly form individual to individual.

### Limitations of the study

The testing was done in a stationary laboratory situation using the Cyclus II ergometer. The absent of a need or possibility to steer the handcycling as well as the able-bodied participant with limited handcycling experience might have influenced test results. This restricts the transferability of the test results onto athletes with spinal cord injuries or other disabilities. A real competitive test setup during a handcycling race, including participants with disabilities would have simulated this more significantly, but tests like that are more or less impossible to be conducted.

Nevertheless, as all grip angles were tested under the same laboratory situation, the results allow claiming relevance for handcycling athletes.

## Conclusion

In the present study there were no differences between three different grip positions (horizontal, vertical, and diagonal) when handcycling at maximum intensity during an incremental test and during a sprint test. There was also no difference in the economy of hand cycling during submaximal loads when each of the three grips was used. The vertical grip was associated with higher lactate concentrations during submaximal handcycling, and may be indicative of reduced efficiency caused by the static (continuous) activation of the working muscles. Further, studies should be conducted to verify these findings during prolonged exercise bouts and in athletes with a spinal cord injury or similar disabilities.

### Conflict of interest statement

The authors declare that the research was conducted in the absence of any commercial or financial relationships that could be construed as a potential conflict of interest.
